# Comparing methods for the analysis of pupillary response

**DOI:** 10.3758/s13428-018-1108-6

**Published:** 2018-10-15

**Authors:** Janice Attard-Johnson, Caoilte Ó Ciardha, Markus Bindemann

**Affiliations:** 1 Department of Psychology, Faculty of Science and Technology, Bournemouth University, Fernbarrow, Poole, BH12 5BB UK; 2 School of Psychology, University of Kent, Canterbury, CT2 7NP UK

**Keywords:** Eye, Pupil, Dilation, Analysis, *z*-scores, Percentage change, Prestimulus baseline correction

## Abstract

Changes in eye-pupil size index a range of cognitive processes. However, variations in the protocols used to analyze such data exist in the psychological literature. This raises the question of whether different approaches to pupillary response data influence the outcome of the analysis. To address this question, four methods of analysis were compared, using pupillary responses to sexually appetitive visual content as example data. These methods comprised analysis of the *unadjusted* (raw) pupillary response data, *z-scored* data, *percentage-change* data, and data transformed by a *prestimulus baseline correction*. Across two experiments, these methods yielded near-identical outcomes, leading to similar conclusions. This suggests that the range of approaches that are employed in the psychological literature to analyze pupillary response data do not fundamentally influence the outcome of the analysis. However, some systematic carryover effects were observed when a prestimulus baseline correction was applied, whereby dilation effects from an arousing target on one trial still influenced pupil size on the next trial. This indicates that the appropriate application of this analysis might require additional information, such as prior knowledge of the duration of carryover effects.

While the pupil of the eye changes size rapidly to protect the cells of the retina from light overexposure (Binda & Gamlin, 2017), it is now becoming clear that pupillary responses also reflect a broad range of higher cognitive processes. These include differences in information-processing load (e.g., Granholm, Asarnow, Sarkin, & Dykes, 1996; Jainta & Baccino, 2010), memory encoding and retrieval (e.g., Goldinger & Papesh, 2012; Otero, Weekes, & Hutton, 2011), target detection (e.g., Privitera, Renninger, Carney, Klein, & Anguilar, 2010), and arousal during the affective processing of visual stimuli (e.g., Bradley, Miccoli, Escrig, & Lang, 2008; Partala & Surakka, 2003). However, across the range of studies utilizing pupillary responses to index cognitive processes, variation exists in the protocols to analyze such data. This raises the question of whether different approaches to pupillary response data influence the outcome of its analysis.

To address this question, we compared four methods for analyzing pupillary responses. For this purpose, we utilized pupillary responses to sexually appetitive visual content as example data. There is converging evidence from an increasing number of studies that pupillary responses provide a measure of arousal during the viewing of sexual content that corresponds to a viewer’s sexual interests. It has been shown, for example, that the pupils of heterosexual men dilate more during the viewing of sexually attractive women than the viewing of men, with the reverse pattern found for homosexual male observers (Attard-Johnson, Bindemann, & Ó Ciardha, 2017; Ó Ciardha, Attard-Johnson, & Bindemann, 2017; Rieger & Savin-Williams, 2012; Watts, Holmes, Savin-Williams, & Rieger, 2017). However, some differences in findings also exist across studies. For example, while some studies have demonstrated sex-specific dilation consistent with sexual orientation (Attard-Johnson & Bindemann, 2017; Attard-Johnson, Bindemann, & Ó Ciardha, 2016, 2017; Hess, Seltzer, & Shlien, 1965; Rieger & Savin-Williams, 2012; Rieger, Savin-Williams, Chivers, & Bailey, 2016; Watts et al., 2017), others have found more generalized or nonspecific pupillary responses across observers for male and female image categories (Aboyoun & Dabbs, 1998; Scott, Wells, Wood, & Morgan, 1967; Snowden, McKinnon, Fitoussi, & Gray, 2017). A number of methodological differences might underlie these diverging results. Notably, however, these studies also differ in their approaches to the analysis of pupillary response and it is not known whether this can contribute to different outcomes.

A summary of studies that have employed pupillary responses as a measure of sexual interest are provided in Table 1. This table shows that, broadly, four different methods have been applied to transforming pupillary responses in the data analysis of studies in this field. Early studies commonly performed analysis on the “unadjusted” area or diameter of the pupil, which was usually expressed in millimeters (Hamel, 1974; Nunnally, Knott, Duchnowski, & Parker, 1967; Scott et al., 1967). This measure is relatively easy to apply, but it has the disadvantage that individuals differ generally in pupil size and, therefore, also in the degree of pupil change that they may exhibit. These factors act as a source of noise that may reduce the statistical power for detecting effects of interest. This problem may be exacerbated further in comparisons of different observer groups, which is common in the study of sexual interest.

**Table 1:** Summary of studies measuring arousal to sexual stimuli, illustrating their different methods for the analysis of pupil size

Study	Pupil transformation	Description
Hess & Polt (1960)	Percentage change in pupil size from prestimulus	Computed percentage pupil size change for each target stimulus from mean pupil size during the 10-s control stimulus preceding each target stimulus
Hess et al. (1965)	Percentage change in pupil size from prestimulus	Computed percentage pupil size change for each target stimulus from mean pupil size during the 10-s control stimulus preceding each target stimulus
Nunnally et al. (1967)	Unadjusted pupil diameter in millimeters	No baseline: Mean pupil diameter taken for each category
Scott et al. (1967)	Experiment 1 Unadjusted pupil diameter in millimeters	No baseline: Mean pupil diameter taken for each category
Scott et al. (1967)	Experiment 2 Unadjusted mean pupil diameter change in millimeters	Difference scores calculated between means for male and female pictures
Atwood & Howell (1971)	Unadjusted mean pupil diameter change in millimeters	Difference scores calculated between means for images of adult females and young females
Hamel (1974)	Unadjusted mean pupil diameter in millimeters	No baseline: Mean pupil diameter taken for each individual stimulus
Dabbs (1997)	Percentage change in pupil size from prestimulus	For each participant, percentage change in pupil size for each stimulus was calculated from the mean pupil size during 10 s of silence prior to auditory stimulus
Aboyoun & Dabbs (1998)	Percentage change in pupil size from the overall mean	For each participant, percentage change in pupil size for each stimulus was calculated from the overall mean across all stimulus categories
Laeng & Falkenberg (2007)	Ratio of the mean pupillary change from the overall mean	Ratio of pupil size change for each stimulus category was calculated from participants’ overall mean across all stimuli
Rieger & Savin-Williams (2012)	*z*-scores of pupillary responses	Computed *z*-scores within each participant for each stimulus, then calculated the mean value across stimulus categories
Rieger et al. (2013)	*z*-scores of pupillary responses	Computed *z*-scores within each participant for each stimulus, then calculated the mean value across stimulus categories
Rieger et al. (2015)	*z*-scores of pupillary responses	Computed *z*-scores within each participant for each stimulus category. Average dilation to neutral stimuli was then subtracted from the mean dilation to each sexual stimulus
Rieger et al. (2016)	*z*-scores of pupillary responses	Computed *z*-scores within each participant for each stimulus, then calculated the mean value across stimulus category
Attard-Johnson et al. (2016)	Percentage change in pupil size from the overall mean	Percentage change in pupil size was calculated for each category from each participant’s overall mean across all stimulus categories
Attard-Johnson et al. (2017)	Percentage change in pupil size from the overall mean	Percentage change in pupil size was calculated for each category from each participant’s overall mean across all stimulus categories
Watts et al. (2017)	*z*-scores of pupillary responses	Computed *z*-scores within each participant for each stimulus category Average dilation to neutral stimuli was then subtracted from the mean dilation to each sexually explicit and nonexplicit stimulus
Attard-Johnson & Bindemann (2017)	Percentage change in pupil size from the overall mean	Percentage change in pupil size was calculated for each category from each participant’s overall mean across all stimulus categories
Finke et al. (2017)	Change in pupil diameter from prestimulus	Change in pupil diameter during stimulus from average value over the 1,000-ms screen prior to stimulus onset
Snowden et al. (2017)	Change in pupil size from prestimulus	Difference scores calculated by subtracting pupil size in each trial from the mean pupillary response obtained during a 2,000-ms prestimulus screen following a 5,000-ms recovery screen

One method for reducing the impact of this individual variation is to standardize pupillary responses. In the sexual interest literature, one method for this purpose is the computation of *z-scores* for individual participants, before calculation of a cross-subject mean (Rieger et al., 2015; Rieger & Savin-Williams, 2012; Watts et al., 2017). An alternative approach to this problem that is also employed in the sexual interest literature is based on the calculation of pupil size change during the viewing of specific stimulus conditions from a baseline. However, the baselines that are employed for this purpose vary across studies. For example, in some studies this baseline reflects mean pupil size across all conditions or stimulus categories in an experiment (Aboyoun & Dabbs, 1998; Attard-Johnson & Bindemann, 2017; Attard-Johnson et al., 2016, 2017; Hess et al., 1965; Laeng & Falkenberg, 2007). The mean pupil size for specific conditions or stimulus categories is then subtracted from this overall mean and represented as the *percentage change* in pupil size from baseline (see, e.g., Attard-Johnson & Bindemann, 2017; Attard-Johnson et al., 2016, 2017). In other studies, a neutral control scene is presented prior to each trial and taken as the baseline (Dabbs, 1997; Hess et al., 1965; Snowden et al., 2017). Combinations of these different methods have also been employed. In some studies, for example, pupil data for conditions displaying sexual content is standardized with *z*-scores *before* subtracting these from either a neutral scene condition or a different type of sexual content condition (see, e.g., Rieger & Savin-Williams, 2012).

It is clear that these different analytical methods to pupillary responses must exert *some* influence on the outcome of data analysis. For example, when the *z*-score or percentage-change approach is employed, any increases or decreases in pupil size for one condition will be in relation to the pupillary responses for all other conditions, and must be interpreted with this in mind. To illustrate, if all conditions in a design elicit sexual arousal that induces pupil dilation (e.g., the viewing of men and women by bisexual observers; see Attard-Johnson et al., 2017), and this dilation is comparable across conditions, then analysis of *z*-scored or percentage-change data will fail to indicate that an increase in pupil size has in fact taken place. By contrast, such pupil dilation will be evident in observers who are aroused by only one of the stimulus categories, as would be the case for heterosexual male observers viewing women but not men. This approach can therefore also make it difficult to compare pupillary responses across different groups of observers.

One method for dealing with pupillary responses under experimental conditions that may resolve this group problem is to apply a *prestimulus baseline correction* (see Bradley et al., 2008; Partala & Surakka, 2003). Here, the raw pupil size during stimulus presentation is subtracted from the raw pupil size during the viewing of a blank prescreen, which is typically shown for between 250 and 1,000 ms immediately prior to stimulus presentation (see Leknes et al., 2012; Snowden et al., 2017; Snowden et al., 2016). This method assumes either that pupillary responses during the blank prescreen will be relatively equal, therefore providing a consistent baseline across trials, or that any variation in pupil size during stimulus presentation that does not arise from the stimulus content itself relates directly to variation in pupil size that was already present during the prescreen. There is evidence for this reasoning. For example, research on pupillary responses while listening to positive, negative and neutral sounds demonstrates that arousal from a previous trial can persist for several s and carry over into the blank prescreen of a subsequent trial (Partala & Surakka, 2003). Similarly, pupillary responses evoked by highly erotic images can continue beyond 1,500 ms after these stimuli are removed from view (Finke, Deuter, Hengesch, & Schächinger, 2017).

However, the question arises of whether such carryover effects are actually maintained beyond the prescreen, once a new stimulus is presented. If not, the subtraction of pupillary response to the prescreen from that of the subsequent stimulus may serve to diminish a genuine effect. Moreover, if carryover effects from previous trials affect the prescreen but not the stimulus period, then the baseline subtraction method could falsely attenuate or exaggerate effects. For example, if the presentation of a sexually attractive female on a preceding trial elicits pupil dilation that carries over into the prescreen of the next trial, then pupil dilation during the subsequent presentation of a stimulus that is also arousing to the observers (i.e., another female) should be reduced by this method of calculation.

Although all of these methods have been applied in the literature (see, e.g., the studies detailed in Table 1), a direct comparison of these different methods of analysis to pupillary response, with the same data set, does not exist. Consequently, it remains unresolved whether these approaches differentially affect the analysis of pupillary responses and, consequently, the interpretation of such data. The present study provides a comparison of these four methods of analysis (i.e., *unadjusted raw pupil scores*, *z-scores*, *percentage of pupillary change*, and *prestimulus baseline correction*) to investigate this question. For this purpose, we reanalyzed a previously published experiment, which examined pupillary responses as an index of sexual interest in pictures of male and female persons (Attard-Johnson et al., 2017), and a second, previously unpublished experiment, using the four analysis methods outlined above.

## Experiment 1

For this study, we reanalyzed the pupillary response data reported in Experiment 1 of Attard-Johnson et al. (2017). This experiment investigated the age and sex specificity of these pupillary responses in hetero-, homo-, and bisexual men during the free viewing of photographs of adult men and women, and prepubescent boys and girls. We summarize all key aspects of the methodology here, but readers are referred to the original study for full details. In the reanalysis of this data, the question of main interest was whether application of different methods of analysis can result in different outcomes. For this purpose, four analytical methods were compared, comprising unadjusted raw pupil scores, *z*-scores, percentage of pupillary change, and prestimulus baseline correction.

### Method

#### Participants

One hundred male students with diverse sexual interests were recruited via an advertisement for the experiment, comprising 59 heterosexual men (mean age 21.6 years, *SD* = 5.6, range = 18 to 50), 20 homosexual men (mean age 24.5 years, *SD* = 7.6, range = 18 to 47), and 21 bisexual men (mean age 21.1 years, *SD* = 2.5, range = 18 to 28). Sexual orientation was confirmed via self-reported sexual interest (see Attard-Johnson et al., 2017).

#### Stimuli and procedure

Participants were invited to take part in an experiment on sexual interests, which involved viewing of photographs of male and female persons of varying ages whilst their eye movements were being recorded. In the experiment, participants were seated in front of the SR EyeLink 1000 eyetracking system, at a viewing distance of 60 cm from the display monitor, which was maintained via a chin rest. The participants’ left eyes were tracked at a rate of 1000 Hz, and calibrated and validated using the standard EyeLink nine-point fixation procedure.

A free-viewing paradigm, in which participants were instructed to view the images as “naturally as they normally would,” was adopted in order to record unconstrained, spontaneous eye movements. The stimuli comprised a total of 20 photographs of adult men and women, and prepubescent boys and girls (five scenes for each of these four categories) on beaches. These targets were depicted in these scenes in swim or leisurewear in similar nonsexual poses. In previous research, the mean ages of these targets were estimated at 26.4 years (*SD* = 2.1) for men, 22.8 years (*SD* = 2.6) for women, 5.7 years (*SD* = 1.1) for boys, and 4.7 years (*SD* = 1.4) for girls (see Attard-Johnson et al., 2016). In addition, a set of control beach scenes with no person content was included (five scenes).

Each trial began with a fixation dot, which allowed for drift correction and ensured that the participants were looking at the center of the screen display during stimulus onset. The experimenter then initiated a gray prescreen, which was displayed for 1 s, via a button press. This was followed by a stimulus display for 10 s, and a gray postscreen for 1 s. In this manner, the 25 stimuli were shown in a random order, which was generated uniquely for each participant by the EyeLink software.

### Results

To analyze the eyetracking data, eye movements were first preprocessed by combining fixations of less that 80 ms with the preceding or following fixations if these fixations fell within half a degree of visual angle (for similar approaches, see, e.g., Attard & Bindemann, 2014; Bindemann, Scheepers, Ferguson, & Burton, 2010). Fixations that fell outside the dimensions of the display monitor or that were obscured by eye blinks were excluded. Pupillary responses for each scene were computed by taking the mean pupil area at each fixation, averaged across the 10-s duration of the target stimulus display (i.e., excluding the pre- and postscreens). The pupil response was then calculated for hetero-, homo-, and bisexual observers across target categories. For this purpose, we compared four separate analyses:

1. The first analysis, termed *unadjusted* pupillary responses, was based on the raw pupil size area, as provided by the EyeLink DataViewer software output.
2. For the second analysis, we computed *z-scored* pupillary responses. For these, each participant’s overall mean pupil size, across all conditions, was subtracted from mean performance in a given experimental condition and divided by the overall standard deviation. As a supplementary analysis, we also computed the *difference scores for z-scored* pupillary responses, by subtracting the *z* scores for each person category from the *z*-scores of the no-person control scenes.
3. The third analysis was based on *percentage change* in pupil size. For this analysis, each participant’s pupil size for each stimulus category was calculated as the percentage change from their overall mean pupillary responses across all scenes, using the formula: 100 − (mean pupil size for condition × 100/overall pupil mean, across all conditions). For the resulting scores, a value of zero indicated no change in pupil size and positive or negative scores reflect relatively larger (dilation) or smaller (constriction) pupil sizes for a stimulus category.
4. The fourth analysis was based on *prestimulus baseline correction*. For this analysis, a size value was calculated on a trial-by-trial basis, by subtracting the mean pupil size during the presentation of a target stimulus from the mean pupil size during the 1-s prescreen. These scores were then averaged across trials in each of the conditions.

**Fig. 1:**
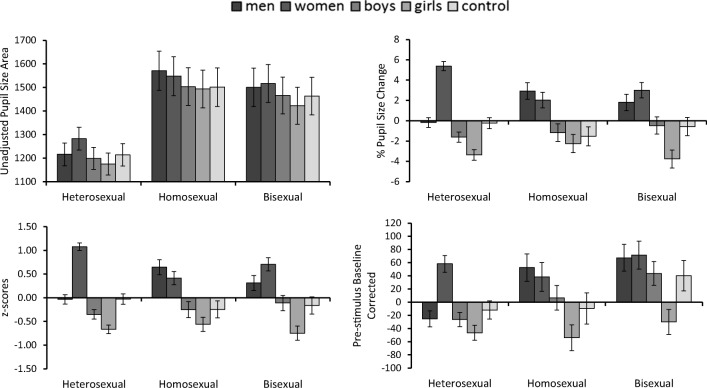
Illustration of the pupillary response patterns for unadjusted pupillary responses (top left), *z*-scored pupillary responses (bottom left), percentages of pupillary change (top right), and prestimulus-baseline-corrected scores (bottom right) for Experiment 1

The pupillary response patterns resulting from these four methods of analysis are illustrated in Fig. 1. These data were analyzed with four separate 5 (category: women, men, girls, boys, control) × 3 (sexual orientation: heterosexual, homosexual, and bisexual) mixed-factor analyses of variance (ANOVAs) for each of these methods, followed up with *t* tests (with alpha adjusted for multiple comparisons). The outcomes of these analyses are reported in Table 2. To facilitate a comparison of the results, this table highlights differences in the outcomes of the four main analysis methods, as well as a supplementary analysis of difference scores for the *z*-scored pupillary responses. These data show that the inferential statistical analysis converged fully, across all comparisons, for the unadjusted and *z*-scored data. The percentage-change data differed from these two methods on one occasion (corresponding to 3.3% of all comparisons), whereby the percentage-change data indicated greater pupil dilation in homosexual men during the viewing of women than of girls using the adjusted alpha level of *p* < .005 (for ten comparisons), but did not cross this threshold for the unadjusted and *z*-scored data.

**Table 2:** Summary of all statistical comparisons for the data analysis of pupillary responses using the four methods of analysis, in Experiment 1 and Experiment 2

	Unadjusted	*z*-scores	Percentage change	Prestimulus baseline corr.	Difference scores with *z*-scores
Experiment 1					
ANOVA	*F*(8, 388) = 3.02^**^	*F*(8, 388) = 3.21^**^	*F*(8, 388) = 3.02^**^	*F*(8, 388) = 3.50^***^	*F*(6, 291) = 4.38^***^
	partial *η*^2^ = 0.06	partial *η*^2^ = 0.06	partial *η*^2^ = 0.06	partial *η*^2^ = 0.07	partial *η*^2^ = 0.08
Heterosexual					
Men vs. women	*t*(58) = 8.90, *p* < 0.001	*t*(58) = 8.95, *p* < 0.001	*t*(58) = 8.82, *p* < 0.001	*t*(58) = 5.41, *p* < 0.001	*t*(58) = 10.14, *p* < 0.001
Women vs. boys	*t*(58) = 8.90, *p* < 0.001	*t*(58) = 8.88, *p* < 0.001	*t*(58) = 8.70, *p* < 0.001	*t*(58) = 6.93, *p* < 0.001	*t*(58) = 10.45, *p* < 0.001
Women vs. girls	*t*(58) = 10.88, *p* < 0.001	*t*(58) = 10.90, *p* < 0.001	*t*(58) = 12.60, *p* < 0.001	*t*(58) = 7.91, *p* < 0.001	*t*(58) = 17.54, *p* < 0.001
Men vs. boys	*t*(58) = 1.90, *p* = 0.07	*t*(58) = 1.84, *p* = 0.07	*t*(58) = 2.03, *p* = 0.05	*t*(58) = 0.96, *p* = 0.92	*t*(58) = 2.26, *p* = 0.03
**Men vs. girls**	***t*** **(58) = 4.26,** ***p*** **< 0.001**	***t*** **(58) = 4.27,** ***p*** **< 0.001**	***t*** **(58) = 4.20,** ***p*** **< 0.001**	***t*** **(58) = 1.85,** ***p*** **= 0.07**	***t*** **(58) = 4.67,** ***p*** **< 0.001**
Girls vs. boys	*t*(58) = 2.37, *p* = 0.02	*t*(58) = 2.39, *p* = 0.02	*t*(58) = 2.03, *p* = 0.05	*t*(58) = 2.07, *p* = 0.04	*t*(58) = 2.07, *p* = 0.04
Men vs. control	*t*(58) = 0.22, *p* = 0.83	*t*(58) = 0.22, *p* = 0.83	*t*(58) = 0.08, *p* = 0.94	*t*(58) = 0.84, *p* = 0.40	
Women vs. control	*t*(58) = 7.27, *p* < 0.001	*t*(58) = 7.30, *p* < 0.001	*t*(58) = 7.11, *p* < 0.001	*t*(58) = 4.60, *p* < 0.001	
Boys vs. control	*t*(58) = 1.57, *p* = 0.12	*t*(58) = 1.54, *p* = 0.13	*t*(58) = 1.58, *p* = 0.12	*t*(58) = 1.23, *p* = 0.22	
**Girls vs. control**	***t*** **(58) = 3.82,** ***p*** **< 0.001**	***t*** **(58) = 3.84,** ***p*** **< 0.001**	***t*** **(58) = 3.73,** ***p*** **< 0.001**	***t*** **(58) = 2.61,** ***p*** **= 0.01**	
Homosexual					
Men vs. women	*t*(19) = 0.99, *p* = 0.34	*t*(19) = 0.99, *p* = 0.34	*t*(19) = 0.67, *p* = 0.51	*t*(19) = 0.64, *p* = 0.53	*t*(19) = 0.81, *p* = 0.43
Women vs. boys	*t*(19) = 2.08, *p* = 0.05	*t*(19) = 2.08, *p* = 0.05	*t*(19) = 2.58, *p* = 0.02	*t*(19) = 1.39, *p* = 0.18	*t*(19) = 2.26, *p* = 0.04
**Women vs. girls**	***t*** **(19) = 2.98,** ***p*** **= 0.008**	***t*** **(19) = 2.98,** ***p*** **= 0.008**	***t*** **(19) = 3.33,** ***p*** **< 0.005**	***t*** **(19) = 4.66,** ***p*** **< 0.001**	***t*** **(19) = 3.47,** ***p*** **< 0.005**
**Men vs. boys**	***t*** **(19) = 3.33,** ***p*** **< 0.005**	***t*** **(19) = 3.33,** ***p*** **< 0.005**	***t*** **(19) = 3.69,** ***p*** **< 0.005**	***t*** **(19) = 1.84,** ***p*** **= 0.08**	***t*** **(19) = 3.39,** ***p*** **< 0.005**
**Men vs. girls**	***t*** **(19) = 3.04,** ***p*** **= 0.007**	***t*** **(19) = 3.04,** ***p*** **= 0.007**	***t*** **(19) = 3.21,** ***p*** **= 0.005**	***t*** **(19) = 4.94,** ***p*** **< 0.001**	***t*** **(19) = 4.24,** ***p*** **< 0.001**
**Girls vs. boys**	***t*** **(19) = 0.48,** ***p*** **= 0.63**	***t*** **(19) = 0.48,** ***p*** **= 0.63**	***t*** **(19) = 0.79,** ***p*** **= 0.44**	***t*** **(19) = 4.11,** ***p*** **< 0.005**	***t*** **(19) = 1.28,** ***p*** **= 0.22**
Men vs. control	*t*(19) = 3.09, *p* = 0.006	*t*(19) = 3.09, *p* = 0.006	*t*(19) = 3.03, *p* = 0.007	*t*(19) = 2.46, *p* = 0.02	
Women vs. control	*t*(19) = 2.31, *p* = 0.03	*t*(19) = 2.31, *p* = 0.03	*t*(19) = 2.55, *p* = 0.02	*t*(19) = 2.12, *p* = 0.05	
Boys vs. control	*t*(19) = 0.14, *p* = 0.89	*t*(19) = 0.15, *p* = 0.89	*t*(19) = 0.34, *p* = 0.74	*t*(19) = 0.96, *p* = 0.35	
Girls vs. control	*t*(19) = 0.35, *p* = 0.73	*t*(19) = 0.35, *p* = 0.73	*t*(19) = 0.44, *p* = 0.67	*t*(19) = 2.59, *p* = 0.02	
Bisexual					
Men vs. women	*t*(20) = 0.72, *p* = 0.48	*t*(20) = 0.73, *p* = 0.48	*t*(20) = 0.75, *p* = 0.46	*t*(20) = 0.07, *p* = 0.95	*t*(20) = 1.25, *p* = 0.22
Women vs. boys	*t*(20) = 2.59, *p* = 0.02	*t*(20) = 2.62, *p* = 0.02	*t*(20) = 2.68, *p* = 0.01	*t*(20) = 1.31, *p* = 0.21	*t*(20) = 2.96, *p* = 0.008
Women vs. girls	*t*(20) = 6.00, *p* < 0.001	*t*(20) = 6.00, *p* < 0.001	*t*(20) = 6.65, *p* < 0.001	*t*(20) = 4.28, *p* < 0.001	*t*(20) = 6.65, *p* < 0.001
Men vs. boys	*t*(20) = 1.65, *p* = 0.11	*t*(20) = 1.67, *p* = 0.11	*t*(20) = 1.55, *p* = 0.14	*t*(20) = 1.31, *p* = 0.21	*t*(20) = 1.39, *p* = 0.18
Men vs. girls	*t*(20) = 3.87, *p* < 0.001	*t*(20) = 3.87, *p* < 0.001	*t*(20) = 3.62, *p* < 0.005	*t*(20) = 3.82, *p* < 0.001	*t*(20) = 4.68, *p* < 0.001
**Girls vs. boys**	***t*** **(20) = 2.30,** ***p*** **= 0.03**	***t*** **(20) = 2.30,** ***p*** **= 0.03**	***t*** **(20) = 1.98,** ***p*** **= 0.06**	***t*** **(20) = 3.67,** ***p*** **< 0.005**	***t*** **(20) = 2.74,** ***p*** **= 0.01**
Men vs. control	*t*(20) = 1.94, *p* = 0.07	*t*(20) = 1.95, *p* = 0.07	*t*(20) = 1.98, *p* = 0.07	*t*(20) = 1.27, *p* = 0.22	
Women vs. control	*t*(20) = 3.15, *p* = 0.005	*t*(20) = 3.20, *p* = 0.005	*t*(20) = 2.91, *p* = 0.009	*t*(20) = 1.38, *p* = 0.18	
Boys vs. control	*t*(20) = 0.10, *p* = 0.92	*t*(20) = 0.10, *p* = 0.93	*t*(20) = 0.06, *p* = 0.95	*t*(20) = 0.18, *p* = 0.86	
Girls vs. control	*t*(20) = 1.91, *p* = 0.07	*t*(20) = 1.92, *p* = 0.07	*t*(20) = 1.98, *p* = 0.06	*t*(20) = 2.88, *p* = 0.009	
Experiment 2					
ANOVA	*F*(2, 97) = 39.56^***^	*F*(2, 97) = 40.01^***^	*F*(2, 97) = 42.63^***^	*F*(2, 97) = 30.97^***^	
	partial *η*^2^ = 0.45	partial *η*^2^ = 0.47	partial *η*^2^ = 0.47	partial *η*^2^ = 0.39	
Heterosexual					
Men vs. women	*t*(58) = 8.04, *p* < 0.001	*t*(58) = 7.58, *p* < 0.001	*t*(58) = 8.32, *p* < 0.001	*t*(58) = 7.58, *p* < 0.001	
Homosexual					
Men vs. women	*t*(19) = 5.50, *p* < 0.001	*t*(19) = 5.58, *p* < 0.001	*t*(19) = 5.91, *p* < 0.001	*t*(19) = 3.79, *p* < 0.001	
Bisexual					
Men vs. women	*t*(20) = 1.29, *p* = 0.21	*t*(20) = 0.44, *p* = 0.67	*t*(20) = 0.93, *p* = 0.36	*t*(20) = 1.68, *p* = 0.11	

Where analysis methods differed in the significance of a comparison, these rows have been highlighted in bold. To adjust for multiple comparisons in Experiment 1, alpha is corrected at *p* < .005 (for ten comparisons). For ANOVAs: ^*^p < .05, ^**^p < .01, ^***^p < .001.

The supplementary analysis of difference scores for the *z*-scored pupillary responses, which were calculated by subtracting the *z*-scores of each person category from the control (landscape) scenes, provides an interesting comparison to these approaches. Inferential analysis of the data converged well with the unadjusted and *z*-scored data, except that it indicated greater pupil dilation in homosexual men during the viewing of women than of girls (see Table 2). This deviation converges with the analysis of the percentage-change data and might reflect that both analytical approaches involve the subtraction of pupil size in a specific condition from a comparison (i.e., mean pupillary responses across all scenes or in the control scenes). In addition, the calculation of difference scores for the *z*-scores also revealed larger pupils in homosexual men during the viewing of men than of girls, whereas this difference was not reliable with the unadjusted, *z*-scored, and percentage-change approaches.

Finally, the prestimulus baseline correction method produced diverging inferential results for seven of the 30 comparisons (23.3% of all comparisons). For example, whereas the other three measures demonstrated significant pupil constriction when heterosexual male observers viewed photographs of girls as compared to adult men, this difference was not significant with the prestimulus baseline correction. On the other hand, this method showed greater constriction when homosexual males viewed boys rather than girls, but these differences were not reliable with the three other methods. Overall, the prestimulus baseline correction method revealed significant differences on three occasions when the other three methods did not, and on one other occasion when the unadjusted and *z*-scored pupil data did not. This analysis also failed to find a significant difference between conditions on three occasions when the other three methods did (see Table 2).

Considering the diverging results for the prestimulus baseline correction from the other methods, we explored whether this analysis might be confounded by dilation elicited during the viewing of a preceding target stimulus, which then carried over into the prescreen period of the next target. To investigate this, pupillary scores for the prescreen period were assigned on the basis of the stimulus content of the preceding trial. A 3 (sexual orientation) × 5 (prior category) mixed-factor ANOVA of this data revealed a main effect of sexual orientation, *F*(2, 97) = 6.22, *p* < .01, partial *η*^2^ (*η*_p_^2^) = .11, whereby pupillary responses of homosexual men were generally larger than those of heterosexual male observers during the prescreen, *t*(77) = 3.35, *p* < .01. The comparisons between bisexual and homosexual, *t*(39) = 0.62, *p* = *.*54, and bisexual and heterosexual, *t*(78) = 2.39, *p* = *.*02, men did not reach significance (with alpha corrected at *p* < .017, *α*/3). In addition, a main effect of prior category, *F*(4, 388) = 2.15, *p* = *.*07, *η*_p_^2^ = .02, and an interaction with sexual orientation, *F*(8, 388) = 1.24, *p* = *.*28, *η*_p_^2^ = .03, were not found. Thus, carryover effects from preceding trials cannot explain the divergent results of the prestimulus baseline correction method in comparison with the other three methods of analysis.

### Discussion

This experiment demonstrated that the four main methods of analysis of pupillary responses under comparison here, as well as the supplementary analysis of *z*-scored difference scores, yielded broadly consistent results. For example, the unadjusted and *z*-scored analyses converged fully across all comparisons, and converged with percentage-change data on 97% of all comparisons. By contrast, the prestimulus baseline correction method converged with the other approaches on only 77% of comparisons. On four of the remaining comparisons, the prestimulus baseline correction method revealed significant differences when the other methods did not, but it also failed to find significant differences between conditions that were apparent with the other approaches in the three remaining comparisons. We conducted a further analysis to explore whether the divergent results of prestimulus baseline correction from the other methods might be related to carryover effects from preceding trials, but we obtained no evidence for this explanation. Overall, this experiment showed broad convergence of results across the different methods of analysis. For example, all methods confirmed the main results of the original experiment, such as greater pupil dilation to pictures of women than of men in heterosexual males, but more matched pupil responses to both target categories in homosexual and bisexual observers.

## Experiment 2

We report a second experiment run to confirm the convergence that was observed across the four methods of analysis in Experiment 1. This experiment applied the same methods to pupillary data from a sexual-appeal rating task. This task utilized a substantially larger stimulus set than had Experiment 1, but it comprised images only of men and women. This methodological difference between the experiments posed an additional question for comparison of the four analysis methods under investigation here, in that the reduced number of stimulus categories might conceal pupillary dilation or constriction effects under some conditions. For example, if the viewing of men and women were to elicit comparable pupil dilation effects in bisexual observers, then the methods of analysis might fail to indicate that an increase in pupil size had, in fact, taken place in either target condition. On the other hand, limiting the number of conditions and increasing the stimulus set in this experiment might also pose an advantage, by increasing the likelihood that systematic carryover effects, if present, would be observed in the prestimulus baseline correction method.

### Method

#### Participants, stimuli, and procedure

The same participants as in Experiment 1 completed this experiment. A total of 36 computer-modified stimuli representing 18 adult men and 18 adult women were used (see Ó Ciardha & Gormley, 2012, 2013). All of these persons were portrayed in black undergarments, similar frontal poses, and similar facial expression. These targets were computer-modified—the faces were morphed composites of multiple faces—and controlled for size and composition. The procedure was identical to that described in the previous experiment, except that participants were instructed to rate the sexual appeal of the people in the images on a 7-point scale, wherein 1 corresponded to *not at all sexually appealing to me* and 7 to *extremely sexually appealing to me*. Thus, each trial commenced with a drift correction, followed by a 1-s prescreen, after which the next target stimulus was presented. Once a response to the target had been made, the stimulus was removed and the trial concluded with a gray postscreen, which was also displayed for 1 s.

### Results

**Fig. 2:**
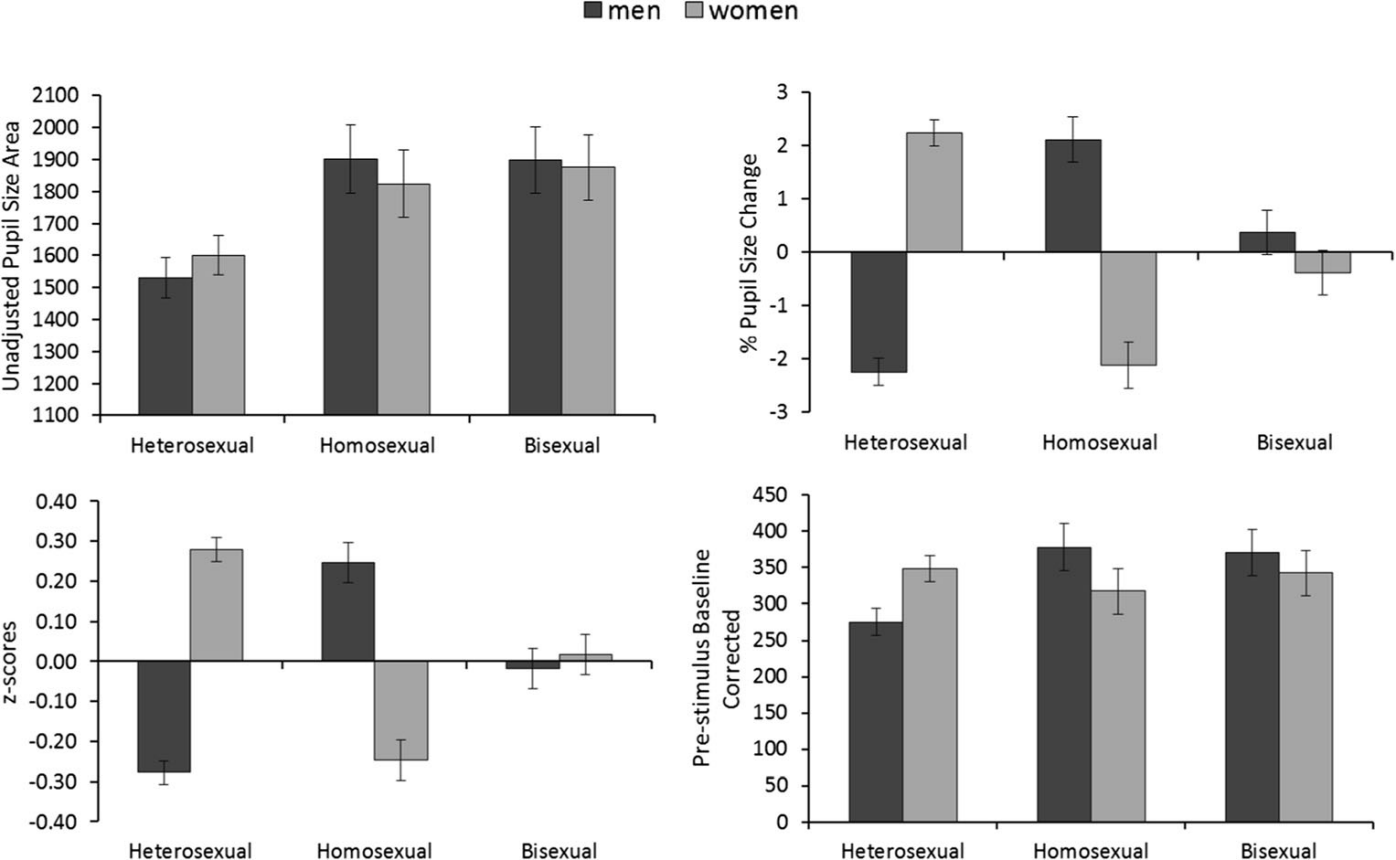
Illustration of the pupillary response patterns for unadjusted pupillary responses (top left), *z*-scored pupillary responses (bottom left), percentages of pupillary change (top right), and prestimulus-baseline-corrected scores (bottom right) for Experiment 2

The eyetracking data were prepared and analyzed in the same manner as in Experiment 1. The pupillary response patterns resulting from the four methods of analysis are illustrated in Fig. 2. These data was analyzed with four separate 3 (sexual orientation: heterosexual, homosexual, and bisexual) × 2 (target sex: male, female) mixed-factor ANOVAs for each of the approaches, which were followed up with *t* tests. The outcomes of these analyses are reported in Table 2. These data show that the inferential analyses converged fully, across all comparisons, for the four analytical methods. Thus, all four methods demonstrated reliably greater dilation during the viewing of women in heterosexual men, during the viewing of men in homosexual men, and no difference during the viewing of male and female targets in bisexual observers.

In addition to the convergence of the analysis methods, we also assessed prestimulus baseline correction further, to examine whether the pupillary responses during the prescreen period were affected by content from the preceding target stimulus. A 3 (sexual orientation: heterosexual, homosexual, and bisexual) × 2 (preceding target sex: male, female) mixed-factor ANOVA did not show a main effect of preceding target sex, *F*(1, 97) = 0.03, *p* = *.*86, *η*_p_^2^ = .00, but revealed an effect of sexual orientation, *F*(2, 97) = 6.45, *p* < .01, *η*_p_^2^ = .12, and an interaction, *F*(2, 97) = 23.86, *p* < .001, *η*_p_^2^ = .33. To further analyze this interaction, paired *t* tests were conducted comparing the preceding target sex categories for each observer group. For heterosexual observers, this analysis revealed that their pupils were larger during the prescreen period when this had been preceded by pictures of adult females rather than males, *t*(58) = 5.53, *p* < *.*001. Homosexual observers showed the opposite pattern, whereby their pupillary responses were larger during the prescreen if this had been preceded by male rather than female stimuli, *t*(19) = 5.60, *p* < .001. In bisexual men, no difference was found for prescreens preceded by male or female stimuli, *t*(20) = 1.32, *p* = *.*20.

**Fig. 3:**
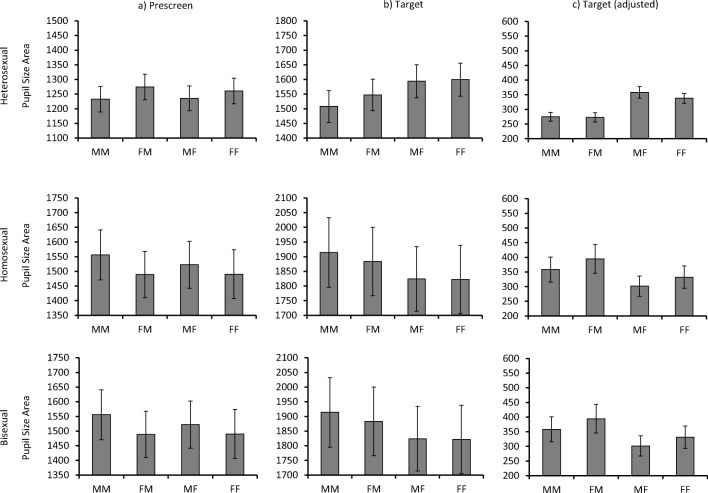
Illustration of pupillary response patterns based on the content of the previous trial (denoted by the first letter) and the current trial (denoted by the second letter; i.e., MF denotes a male target on the previous trial, followed by a female target on the current trial). These are presented for the prescreen (**a**), target screen (**b**), and target screen after prestimulus baseline correction was applied (**c**)

Next we explored whether these differences in pupillary responses during the prescreen period might have also influenced the pupillary responses to the target stimuli when the prestimulus baseline correction method was applied. For this analysis, we computed the pupillary responses for each trial on the basis of the target sex on the previous trial (male or female) and the target sex on the current trial (male or female). This resulted in a total of four conditions, combining a preceding male target and current male target (MM), a preceding female target and current male target (FM), a preceding male target and current female target (MF), and a preceding female target and current female target (FF). Figure 3 illustrates the pupillary response patterns during presentation of the prescreen (Fig. 3a), and the current target (Fig. 3b), and the current target after the prestimulus correction is applied to this stimulus (Fig. 3c). Table 3 summarizes a series of 2 (current-trial target sex: male, female) × 2 (preceding-trial target sex: male, female) within-subjects ANOVAs, which were conducted separately for heterosexual, homosexual, and bisexual observers for the prescreen and target data.

**Table 3:** A summary of the statistical analyses for pupillary responses during the prescreen, the target screen, and the target screen following application of the prestimulus baseline correction, based on the content of the previous trial and the current trial

	Prescreen	Target	Target (Adjusted)
Heterosexual			
Previous category	*F*(1, 58) = 30.55, *p* < .001	*F*(1, 58) = 11.93, p < .01	*F*(1, 58) = 2.91, *p* = .09
Current category	*F*(1, 58) = 0.71, *p* = .40	*F*(1, 58) = 70.50, p < .001	*F*(1, 58) = 61.52, *p* < .001
Previous × Current	*F*(1, 58) = 2.71, *p* = .11	*F*(1, 58) = 8.32, p < .01	*F*(1, 58) = 3.21, *p* = .08
Homosexual			
Previous category	*F*(1, 19) = 30.21, p < .001	*F*(1, 19) = 1.81, *p* = .19	*F*(1, 19) = 7.42, *p* < .05
Current category	*F*(1, 19) = 2.57, *p* = .13	*F*(1, 19) = 27.35, *p* < .001	*F*(1, 19) = 14.23, *p* < .01
Previous × Current	*F*(1, 19) = 1.52, *p* = .23	*F*(1, 19) = 1.65, *p* = .22	*F*(1, 19) = 0.12, *p* = .74
Bisexual			
Previous category	*F*(1, 20) = 1.61, *p* = .22	*F*(1, 20) = 0.01, *p* = .93	*F*(1, 20) = 1.48, *p* = .24
Current category	*F*(1, 20) = 0.48, *p* = .50	*F*(1, 20) = 1.67, *p* = .21	*F*(1, 20) = 3.29, *p* = .08
Previous × Current	*F*(1, 20) = 0.80, *p* = .38	*F*(1, 20) = 2.79, *p* = .11	*F*(1, 20) = 0.04, *p* = .85

#### Pupillary responses during prescreen

During presentation of the prescreen, pupillary responses revealed a pattern that was consistent with observers’ sexual orientation and the sex of the preceding target. Thus, heterosexual observers showed an effect of previous category due to more dilated pupils after the viewing of a female person, whereas homosexual observers exhibited greater dilation after the viewing of men, and bisexual observers showed no difference between the male and female target conditions (see Table 3 and Fig. 3a). This pattern demonstrates clear carryover effects, whereby the target stimuli from the preceding trial still influenced pupillary responses during the prescreen period of the next trial. As one would expect, these effects were not affected by the sex of the target presented immediately after the prescreen (i.e., the current target).

#### Unadjusted pupillary responses during presentation of the current target

Pupillary responses during the presentation of the current targets were also consistent with observers’ sexual orientation. For example, homosexual observers demonstrated an effect of current category, due to larger pupils during the viewing of men than of women, whereas bisexual observers displayed comparable pupil sizes for both stimulus categories. In both of these groups of observers, these effects do not appear to have been influenced by the stimulus content of the preceding trial (see Table 3 and Fig. 3b). A slightly different pattern emerged in heterosexual observers, who displayed concurrent effects of previous stimulus category, with larger pupils *after* the presentation of female as compared to male pictures, and of current stimulus category also, with larger pupils *during* the presentation of female as compared to male pictures.

In addition, an interaction of these factors was found. A series of four paired-sample *t* tests (with alpha corrected at *p* < .013, *α*/4) showed that the pupils were larger during the viewing of female than of male targets, when these were preceded by either a male target, *t*(58) = 8.50, *p* < .001, or a female target, *t*(58) = 5.16, *p* < .001, on the previous trial. In addition, pupillary responses were comparable for female targets irrespective of whether these had been preceded by male or female targets on the previous trial, *t*(58) = 0.67, *p* = *.*51. By contrast, male targets elicited larger pupillary responses if they had been preceded by a female rather than by a male target, *t*(58) = 4.22, *p* < .001. Thus, in heterosexual men, pupil size was consistently larger during the viewing of a person of the arousing sex (i.e., female targets in the MF and FF conditions), and *also* during the viewing of a person of nonarousing sex when this followed the viewing of a person of the arousing sex (i.e., male targets following female targets in the FM condition).

Overall, this analysis shows that the stimulus content of both a current trial and a preceding trial can affect observers’ pupillary responses. These concurrent effects were reliable only in heterosexual observers, though we note that the patterns of pupillary responses in homosexual and bisexual observers were numerically consistent with this observation. It is possible that the differences in inferential statistics between these groups relate to the sample size, which was almost three times larger for heterosexual than for bisexual and homosexual observers (*N* = 59 vs. 21 and 20).

#### Prescreen adjusted pupillary responses during presentation of the current target

The final part of this analysis explored the pupillary response patterns to target stimuli when these were adjusted through subtraction of the prescreen baseline. We began by considering the group of bisexual observers, who did not reveal any effects of either current-trial target sex or preceding-trial target sex (see Table 3 and Fig. 3c). This is consistent with the separate analysis of the prescreen data for this group, as well as with the unadjusted target data, both of which indicated that these observers produced equivalent pupillary responses to male and female stimuli.

Heterosexual observers showed only an effect of current-trial target sex for the prestimulus adjusted pupillary responses, due to greater pupil dilation during the viewing of female than of male targets. In the context of an effect of preceding-trial target sex during the prescreen and for the unadjusted current-trial target data, this indicates that the subtraction of the former from the latter helped attenuate carryover effects in the current-target data.

However, the data for homosexual observers indicated that this subtraction adjustment method can also produce a different outcome. During the prescreen period, these observers showed an effect of preceding-trial target sex, but this same effect was not reliable in the unadjusted pupillary responses to the target. As a consequence, subtracting these measures in order to create the adjusted pupillary responses to the target stimuli resulted in an effect of previous target that actually indicated *greater* pupil dilation after the viewing of women than of men. This pupillary response effect is nonsensical in homosexual male observers, in the sense that it suggests greater sexual interest in female than in male targets. This points to overcompensation for the prescreen effects by this method of analysis.

### Discussion

This experiment converged with Experiment 1 by demonstrating that the four methods of analysis for pupillary responses yielded broadly consistent results. In fact, with the reduced design of the present experiment, which comprised only stimuli of adult males and females, the analysis of inferential statistics converged fully for all four methods. This reduced design has some limitations, in that it can conceal pupillary dilation effects in observers that are aroused by both categories, which appears to have been the case with bisexual observers here across all four methods of analysis. On the other hand, the more restricted design of this experiment also allowed us to probe the effect of the prestimulus adjusted pupillary response method in greater detail. First, this analysis showed clear carryover effects from the targets of one trial onto the prescreen of the next trial. These effects are remarkable insofar that they survived a posttarget screen and a drift correction between trials, indicating that such carryover may be fairly resistant to extinction. There was also some evidence that these carryover effects can still influence pupillary responses to the next (i.e., the current) target stimulus, though this effect was only reliable in the largest observer group, comprising heterosexual males. Crucially, application of the prescreen adjustment appeared to remove this carryover effect from the pupillary response data during current target presentation, which confirmed that this method of analysis can eliminate such effects.

However, Experiment 2 also indicated some potential problems with this approach, in that homosexual observers demonstrated a carryover effect from the target of one trial to the prescreen of the next trial. However, this carryover was no longer evident in the unadjusted data for the subsequent targets. In this case, subtraction of the pupillary responses at prescreen from those of the target produced the paradoxical finding of reduced pupil dilation following the presentation of a sexually arousing target on the previous trial. This finding highlights a potential problem with the prestimulus baseline correction, whereby this approach should perhaps be applied only when the time course of carryover effects is known accurately. Otherwise, there is a risk that a carryover effect at prescreen will not persist during subsequent target presentation, leading to an overadjustment when the prestimulus baseline correction is applied.

We make this suggestion cautiously, because we note that these carryover effects and their influence on pupillary response to the target were only evident when this was explicitly coded in the analysis. When the analysis was focused solely on the current target (even when prestimulus baseline correction had been applied), all four methods under investigation revealed outcomes in this experiment equivalent to those in Experiment 1.

## General discussion

Investigations into the measurement of pupillary responses vary in their approach to data analysis, but it is unclear whether this affects their outcomes. This study compared four different methods for analyzing pupillary responses, using data from the measurement of sexual interest as an example. These methods comprised *unadjusted pupil scores*, *z-scored pupillary responses*, *percentage change in pupil size*, and *prestimulus baseline corrected* pupil scores. Across two experiments, the methods based on unadjusted pupil scores, *z*-scored pupillary responses, and percentage change in pupil size produced closely comparable results. These three measures converged fully in Experiment 1 and 2, except for one occasion, in which the outcome of the percentage-change analysis differed from the other two methods (corresponding to 3.3% of all comparisons in Exp. 1). In terms of the *z*-scored pupillary responses and percentage of pupillary change, this might reflect the fact that these two methods operate in a similar way, whereby scores are standardized within each participant based on their mean response to all stimuli (in case of *z*-scores) or all categories (in case of percentage pupillary change). However, the fact that these two methods also converge with unadjusted pupil scores demonstrates that such standardization does not change the qualitative outcome of data analysis, at least for the data set under analysis here.

The prestimulus baseline correction differs from the other methods in introducing a further factor, which reflects pupillary responses to an additional stimulus (i.e., the blank prescreen presented immediately prior to a target). The effect of this additional factor is somewhat unpredictable. In the reduced design of Experiment 2, the prestimulus baseline correction method converged fully with the three other measures. However, this method of analysis was the least consistent measure in Experiment 1, wherein this analysis diverged from the other three methods on seven occasions (comprising 23% of comparisons). In three of these cases, the results switched from significance to nonsignificance, whereas four other cases showed the reverse pattern (see Table 2).

The discrepancy in pupillary patterns could be due to a number of factors, but one of these may reflect carryover effects from the target stimulus of the preceding trial onto the prescreen period of next trial. This could result in a systematically variable baseline across trials, which then influenced response calculation for subsequent targets. To examine this, we analyzed pupil size for the prescreen period according to the type of target that was presented on the preceding trial. For Experiment 1, this failed to reveal systematic effects of prior target category, which suggests that carryover effects from the preceding trials cannot explain the divergent results of the prestimulus baseline correction from the other three methods. For Experiment 1, however, these conclusions are tempered by the experimental design, which consisted of five stimulus categories (female, male, girls, boys, and no-person control scenes), each comprising of only five images. This leads to 25 possible category combinations across successive trials, in an experiment comprising only 25 trials, which therefore represents an inappropriate design to assess carryover effects systematically.

The much more constrained design of Experiment 2, which consisted of fewer stimulus categories (i.e., male and female adults) and more stimuli (18 per category), presented an opportunity to probe carryover effects more systematically. This provided several insights. Firstly, this experiment showed clear and systematic carryover effects from one trial to the next, whereby the target’s sex in the preceding trial influenced pupil size during the prescreen period of the next trial consistent with observers’ sexual orientation (see also Partala & Surakka, 2003). Secondly, this experiment adds to the existing evidence that pupillary responses to one target can still affect responses to a subsequent target (Hyönä, Tommola, & Alaja, 1995). However, and thirdly, these carryover effects from one target to the next were also somewhat inconsistent, in the sense that these were observed with heterosexual but not homosexual observers.

This discrepancy across observer groups highlights another important aspect to consider when applying this method of analysis. In the case of the heterosexual observers, subtracting pupillary responses during the prescreen from those of the subsequent target seems sensible, as both appeared to be subject to the same carryover effect (from the target of the preceding trial). Subtracting one measure from the other therefore provides a sensible method to eliminate these analogue carryover effects. By contrast, in homosexual observers, who displayed carryover effects onto the prescreen but not the subsequent target, the subtraction of pupillary responses at prescreen from that to the target produced a paradoxical finding, whereby this adjustment produced a reduction in pupil dilation following the presentation of a sexually arousing target on the previous trial. The contrast between these findings for heterosexual and homosexual observers here is potentially problematic in that it demonstrates that the prestimulus baseline correction method can lead to an overadjustment if a carryover effect at prescreen does not persist during subsequent target presentation.

One method to control for the potential unpredictability of these carryover effects would be to employ a poststimulus “recovery” screen in order to allow the pupil size to return to a natural baseline level (Finke et al., 2017; Snowden et al., 2017). However, in the measurement of sexual interest—with pupillary response, at least—it is unclear how long such a recovery period must be. In the experiments reported here, each stimulus was followed by a gray postscreen for a duration of 1,000 ms, followed by a drift correction ranging from 500 to 1,000 ms, and then the gray 1,000-ms prescreen. Carryover effects persisted nonetheless. Other recovery durations have been reported across studies—for example, intervals of up to 5 s when viewing affective and sexual stimuli (Partala & Surakka, 2003; Snowden et al., 2017; Snowden et al., 2016), or of 10 s and above (Dabbs, 1997; Finke et al., 2017; Hess & Polt, 1960; Hess et al., 1965). A practical disadvantage of such long intervals is that it can increase the duration of experiments greatly. It might impact also on the attention and engagement of participants.

On the other hand, the *minimum* duration required for an evoked pupil dilation response to return to baseline is not currently known, and it may be dependent on the underlying cognitive processes that are recruited by specific tasks. It is possible, for example, that the pupil dilation elicited by sexual arousal may differ in strength, sustained dilation, and lasting duration from similar responses elicited by other affective (e.g., Partala & Surakka, 2003; Snowden et al., 2016) and emotional (e.g., Siegle, Steinhauer, Carter, Ramel, & Thase, 2003) processes, recognition memory (e.g., Goldinger & Papesh, 2012; Heaver & Hutton, 2011), and cognitive load (Jainta & Baccino, 2010). Therefore, the best approach to the application of a prestimulus baseline correction may require knowledge of the duration of carryover effects in the first place, to determine whether this method should be applied in the context of a specific experimental design.

We raise these points for consideration by other researchers, but we do not wish to overstate the contribution of such carryover effects from the present experiments. Ultimately, the methods under comparison here produced highly similar results. Moreover, despite the systematic carryover effects that were observed in Experiment 2, this experiment still produced results equivalent to those from the other analysis methods when preceding-trial data were not coded as a factor in the analysis. Thus, with the data sets that were employed here to compare these methods, the four different analyses led to near-identical conclusions. It remains to be seen whether the equivalence of these methods of analysis will hold with other populations, different types of stimuli, and in the study of other cognitive processes. At this point, however, the present findings are positive, in that they suggest that the range of approaches that are employed in the psychological literature to analyze pupillary response data in studies on sexual orientation and sexual interest should not fundamentally influence the outcome of this analysis.
